# Sensitive detection of circular DNAs at single-nucleotide resolution using guided realignment of partially aligned reads

**DOI:** 10.1186/s12859-019-3160-3

**Published:** 2019-12-12

**Authors:** Iñigo Prada-Luengo, Anders Krogh, Lasse Maretty, Birgitte Regenberg

**Affiliations:** 1 Department of Biology, University of Copenhagen, Ole Maaløes Vej 5, DK-2200 København N, Denmark; 2 Department of Computer Science, University of Copenhagen, Universitetsparken 1, DK-2100 København Ø, Denmark; 3 Department of Molecular Medicine, Aarhus University, Palle Juul-Jensens Boulevard 99, DK-8200 Aarhus N, Denmark

**Keywords:** Structural variation, Next generation sequencing, circRNA, eccDNA, ecDNA, Extra chromosomal circular DNA

## Abstract

**Background:**

Circular DNA has recently been identified across different species including human normal and cancerous tissue, but short-read mappers are unable to align many of the reads crossing circle junctions hence limiting their detection from short-read sequencing data.

**Results:**

Here, we propose a new method, Circle-Map that guides the realignment of partially aligned reads using information from discordantly mapped reads to map the short unaligned portions using a probabilistic model. We compared Circle-Map to similar up-to-date methods for circular DNA and RNA detection and we demonstrate how the approach implemented in Circle-Map dramatically increases sensitivity for detection of circular DNA on both simulated and real data while retaining high precision.

**Conclusion:**

Circle-Map is an easy-to-use command line tool that implements the required pipeline to accurately detect circular DNA from circle enriched next generation sequencing experiments. Circle-Map is implemented in python3.6 and it is freely available at https://github.com/iprada/Circle-Map.

## Background

Circular DNA from all parts of the genome has recently been discovered in model organisms [1, 2], as well as both normal [3, 4] and cancerous human tissues [5, 6] using short-read sequencing based approaches. The primary circular DNA signals in short read data are “discordantly” mapped paired-end reads and “split-reads” crossing the circle breakpoint. Yet standard short read aligners do not reliably detect the latter signal, which is vital for determining the exact circle coordinates, as only alignments that are collinear with the reference sequence are typically considered. Reads that cross the breakpoint of a circular DNA will therefore typically be reported either as two or more separate alignments (a primary and some supplementary alignments) when the read has long “anchors” on both sides of the breakpoint or with a major aligned part and a minor, “soft-clipped” (i.e. unaligned) part. While some tools have used short read aligners to detect the circular DNA signals [2, 4], these methods are unable to detect split reads signals shorter than 19 base-pairs due to the constraints imposed by the standard short read alignment algorithms.

**Fig. 1:**
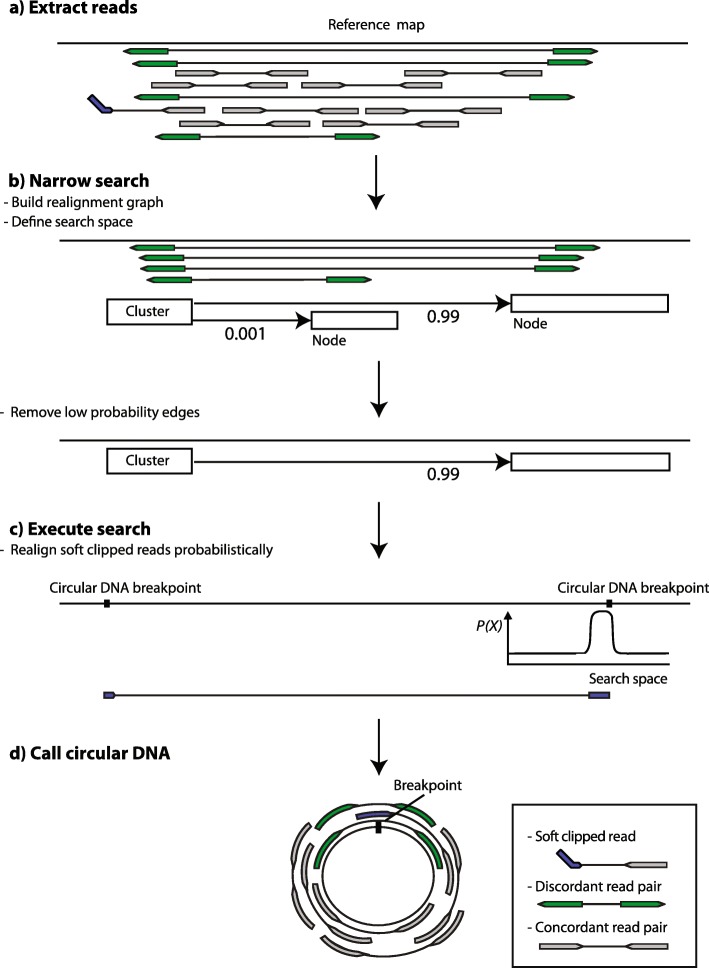
Circle-Map read realignment strategy. **a** Reads are mapped to the reference genome and discordantly aligned reads (green) and alignments containing soft clips (blue) are extracted; concordantly aligned reads (grey) are ignored. **b** Using the extracted reads, a graph of putative breakpoint connections between genomic regions is constructed and used as a prior to narrow down the genomic search space for realigning soft clipped reads. **c** Non-aligned parts of the soft clipped reads are realigned probabilistically using the breakpoint graph as guide. **d** Evidence from split-reads and discordant reads are combined to create the final circle calls together with information about concordant, split-read and discordant read coverage for each circle

We have developed a new bioinformatic method, Circle-Map, to accurately identify circular DNA breakpoints. The overall idea in this method is to use information from discordantly mapped paired-end reads as a *prior* for realigning the soft clipped parts of breakpoint reads, which in turn should allow for more accurate detection of circle breakpoints. Circle-Map consists of three main steps: 1) circular DNA candidate read identification, 2) breakpoint graph construction and 3) soft-clip realignment (Fig. 1).

## Implementation

### Candidate read identification

The Circle-Map workflow begins by performing an initial pass through a query name sorted alignment file to extract reads potentially originating from circular DNA (“circular DNA candidate reads”): discordant read pairs, soft-clipped reads and hard clipped reads (Fig. 1a). For every read pair, Circle-Map labels the pair as discordant if the reads have aligned in opposite orientation and the leftmost mapping position for the second read is smaller than the leftmost mapping position of the first read. If the read pair is not extracted as discordant, Circle-Map will independently extract read pairs with any unaligned bases (soft-clipped and hard clipped).

### Realignment graph construction

Circular DNA identification begins by identifying clusters of candidate reads that are less than *K* nucleotides apart (discordant, soft-clipped and hard-clipped reads). For every cluster, we construct a weighted graph *G = (N,E)* with nodes *N = {n*_*0*_*,…,n*_*i*_*}*, which correspond to regions containing at least one breakpoint of unknown exact location, and edges *E = {e*_*0,*_ *…, e*_*i*_*}*, which correspond to circle variants represented as connections between breakpoint regions (Fig. 1b). For every node n_i_, we obtain the set of edges connected to n_i_ by connecting the alignment positions of the reads belonging to node n_i_ with the alignment positions of their mates (for the discordant read pairs) and supplementary alignments (for the hard-clipped and soft-clipped reads). We then estimate edge weights ${w}_{e_i}$ for every edge e_i_ using the mapping quality scores of the discordant mates and supplementary alignments that support the edge using:

$$ {w}_{e_i}=\frac{\sum_{x\in {e}_i}{10}^{\frac{-{Q}_x}{10}}}{\sum_{e_i\in E}{\sum}_{x\in {e}_i}{10}^{\frac{-{Q}_x}{10}}} $$

where *x* indicates a discordant read or supplementary alignment that supports e_i_ and *Q*_*x*_ indicates the phred scaled mapping quality of read *x*. Edges with weights below 0.01 are removed before the realignment step as it is very unlikely that the edges with low weight contain the true circular DNA breakpoint.

To define the final search space for the realignment step, node intervals are extended to [−*μ* - 5σ, *μ* + 5σ], where *μ* and σ denote the mean and standard deviation of the insert size distribution estimated from concordantly mapped reads, to ensure that nodes originating from discordant reads will contain the breakpoint.

### Soft-clip realignment

In order to realign non-aligning bases of soft-clipped reads and obtain the circular DNA breakpoints at nucleotide resolution, we realign the soft-clipped parts of the reads probabilistically to the pruned realignment graph (Fig. 1c). We build a probabilistic model of the alignment using a Position-Specific Scoring Matrix (PSSM) that takes into account alignment mismatches and indels caused by sequencing errors.

Our algorithm begins by obtaining all possible alignments to the realignment graph using the infix Myers bit-vector algorithm as implemented in the Edlib library [7, 8]. In practice, we obtain the top scoring alignments to the graph by aligning the read and then masking the alignment coordinates, in order to keep searching for suboptimal alignments. For every alignment, we construct a PSSM using the following error model for matches and mismatches:

$$ \left\{\begin{array}{c}1-{p}_e\kern0.5em {a}_i={g}_j\\ {}\\ {}\frac{p_e}{3}\kern2.5em {a}_i\ne {g}_j\end{array}\right. $$

where *a*_*i*_ and *g*_*j*_ denote the identity of the bases on the read and the genome, respectively, and *p*_*e*_ indicates the probability of the base being sequenced wrong as determined from the base quality scores. Next, we compute the log-odds score for every base in the read by diving *P(a*_*i*_*|g*_*i*_*)* by the frequency of base *g*_*j*_ in the realignment graph, denoted as *q(g*_*j*_*)*:

$$ {S}_{a_i}={\mathit{\log}}_2\frac{P\left({a}_i|{g}_j\right)}{q\left({g}_j\right)} $$

A score for the read is calculated by summing over the base scores in the PSSM:

$$ {S}_a={\sum}_i{S}_{a_i} $$

We then add an additional penalty to the PSSM score in order to account for insertions and deletions using an affine gap scoring scheme (adapted from [9]) to yield the final alignment log-odds score:

$$ {S}_x={S}_a+{\mathit{\log}}_2\left(\ {2}^{-p-\left(a-1\right)e}\right) $$

where *p* indicates the insertion and deletion penalty, *a* indicates the length of the event and *e* is the affine gap penalty. Finally, Circle-Map converts the estimated alignment scores to alignment probabilities using:

$$ P(x)=\frac{2^{S_x}}{\sum_{x\prime }{2}^{S_{x\prime }}} $$

where the summation in the denominator runs over all possible alignments in the realignment graph. We only consider soft-clipped reads as realigned if the probability for its high scoring alignment is greater than 0.99, which should maintain the number of incorrect realignments low. The final realignment coordinates define putative circle coordinates (Fig. 1d). We consider a circular DNA as called if it is supported by a minimum of two breakpoint reads and contains at least one split read. Circle-Map reports all called circles with coordinates (chromosome, start and end), number of supporting reads (discordant and soft-clipped) and concordant coverage metrics (Material and methods).

## Results

We evaluated the performance of Circle-Map on both simulated data and real, circle-enriched data from human muscle tissue [3]. We compared the performance of Circle-Map with two split read based methods for the detection of DNA and RNA sequences of circular nature: Circle_finder [4], a circular DNA detection method, and CIRCexplorer2 [10], which has been ranked as one of the best choices for circular RNA detection [11]. Importantly, CIRCexplorer2 does not use splice-site or reference transcriptome information in the split read detection step. We further included Circle-Map without the realignment step as a baseline.

**Fig. 2:**
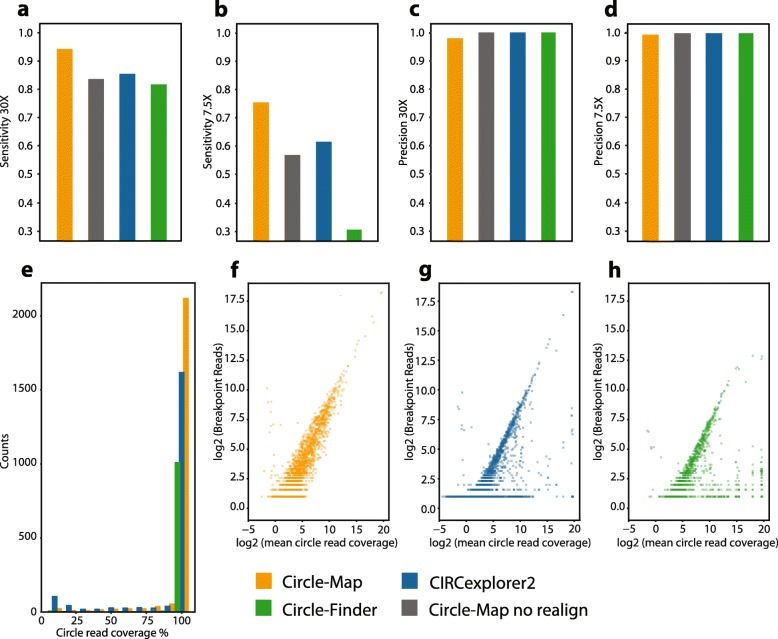
Evaluation of the circular DNA detection methods. **a-d** Circle-Map (orange), CIRCexplorer2 (blue), Circle_finder (green) and Circle-Map with no realignment (grey) were evaluated on simulated circular DNA datasets with varying sequencing depths (**e**-**g**) and on real circle enriched data from human muscle. **a** Sensitivity at 30X and **b** 7.5X measured as the number of called circles found in the simulation set divided by the total number of simulated circles. Precision at (**c**) 30X (**d**) and 7.5X measured as the number of correctly called circles divided by the total number of called circles, true and false. **e** Histogram with the percentage of bases covered by sequencing reads for every circular DNA detected. The number of breakpoint reads (e.g. split and discordant reads) relative to the mean sequencing coverage within the circular DNA coordinates for **f** Circle-Map, **g** CIRCexplorer2 and **h** Circle_finder

**Fig. 3:**
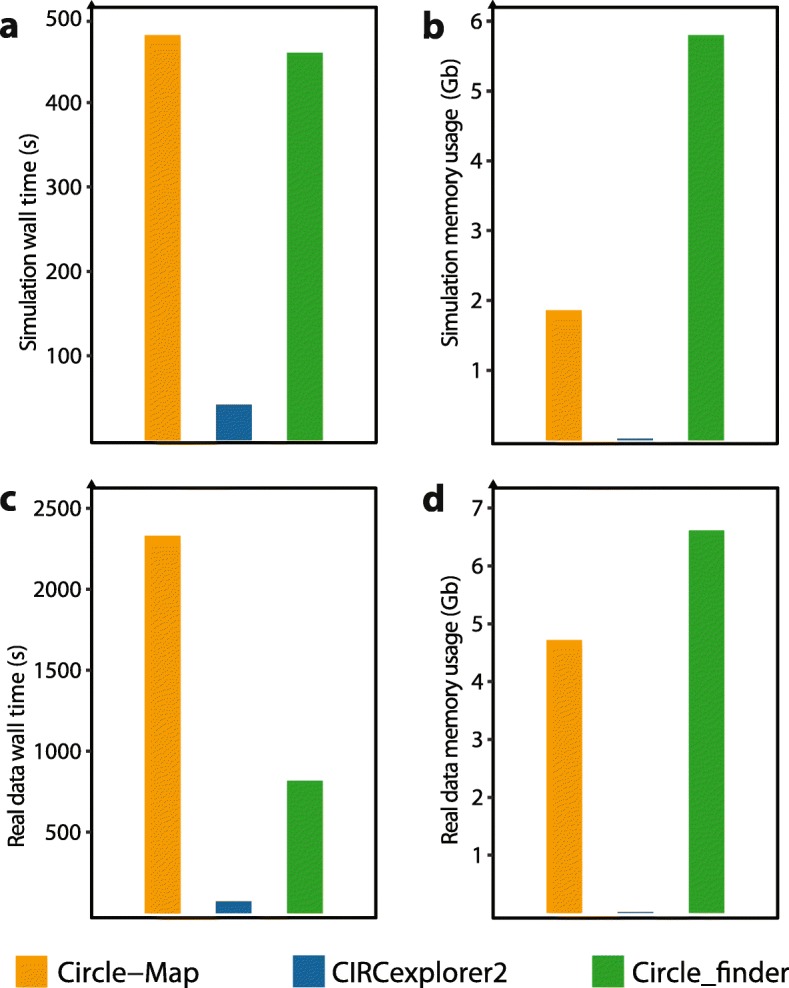
Evaluation of the computation time and memory usage of the circular DNA detection methods. The runtimes and maximum memory usage of Circle-Map (orange), CIRCexplorer2 (blue) and Circle_finder (green) were evaluated on simulated (**a-b**) and real (**c-d**) circular DNA datasets. **a** Wall time and **b** maximum memory usage on the 30X simulated dataset. **c** Wall time and **d** maximum memory usage on the real circular DNA enriched dataset from human muscle

In the simulation benchmark, we simulated both high (30X) and low (7.5X) coverage sequencing of 13,097 circular DNAs across different lengths (range: 150–10,000 nts) and including SNVs and indels based on reference data from the 1000 genomes projects [12]. To evaluate performance, we measured sensitivity, defined as the number of called circles present in the simulation set and precision defined as the fraction of correctly called circles found on the simulated set. On high coverage data (30X), Circle-Map attained a sensitivity of 0.943 by detecting 12,351 of the simulated circles and outperformed CIRCexplorer2 and Circle_finder by 12 and 17 percentage points, respectively (Fig. 2a). A high sensitivity was also found on low coverage data (7.5X) where Circle-Map detected 75% of all circles in contrast to CIRCexplorer2 that detected 61% of the circles, and Circle_finder, that only detected 30% of the circles (Fig. 2b). All three methods attained precision higher than 0.97 on both high and low coverage data sets (Fig. 2c and d). To gain more insights into the performance of Circle-Map on short circles, we simulated reads at 30X from 5384 very short circles with lengths ranging from 150 to 350 nts including indels and SNVs as described above. Again, Circle-Map performed well by achieving a sensitivity of 0.89 and a precision of 0.98 on short DNA circles. Importantly, the accuracy gain does not come with substantially increased computational costs (Fig. 3). Our programs longest runtime was less than 40 min with a maximum memory footprint of 5 gigabytes.

**Fig. 4:**
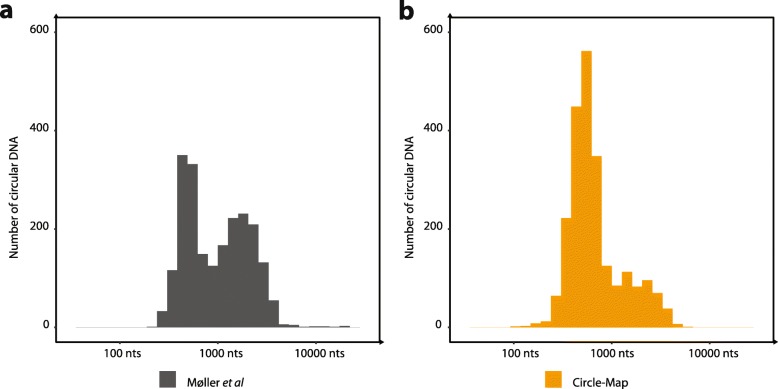
Size distribution of the DNA circles found the circular DNA enriched muscle dataset. Evaluation of the circular DNA size distributions found by the method described by Møller et al.*,* from a previous study [3] (**a**) and the size distribution found by Circle-Map (**b**)

We assessed the performance on real data using a paired-end sequencing dataset from a previous study [3], where circular DNA had been enriched from human muscle tissue by removal of linear DNA and amplification of the circular DNA prior to sequencing. As we lack a ground truth on real data, we instead used the fact that the data were enriched for circles and hence that most coverage from concordantly and contiguously aligned reads (i.e. non split-reads) across the genome should originate from circular DNA. We used the circle coverage fraction as an orthogonal proxy for correctness of the circle. Circle-Map detected 2318 DNA circles with similar sizes to the ones found in the original study [3]. From those, 2170 circles had > 80% coverage while only 148 potential circles had a coverage less than 80% (Fig. 2e). In comparison, CIRCexplorer2 and Circle_finder detected substantially less high coverage circles than Circle-Map (Fig. 2e). CIRCexplorer2 detected only 1655 DNA circles with a coverage > 80% and also detected a larger number of circles with coverage less than 20% (289 circles). Circle_finder, detected less than half of the high coverage circles detected by Circle-Map (1013 circles) while keeping the number of low coverage circles small (16 circles). Compared to the original published circles from human muscle tissue, we found that Circle-Map gave similar circle size ranges with minor differences in the data distribution (Fig. 4).

Finally, we investigated the relationship between the number breakpoint reads (i.e. split and discordant reads) and the concordant reads within the circle coordinates. We reasoned that in circle enriched data, the number of breakpoint reads should correlate with the abundance of the concordant reads. Hence, strong disagreements between breakpoint and concordant read counts should be indicative of erroneous circle detections and a plot of the number of breakpoint reads against the circle mean coverage can serve as a diagnosis tool to verify the agreement between breakpoint and concordant reads. Circle-Map obtained a Pearson’s correlation coefficient of 0.868 and showed a strong concordance between breakpoint reads and concordant reads (Fig. 2f). In contrast, CIRCexplorer2 (Fig. 2g) and Circle_finder (Fig. 2h) achieved correlation coefficients of 0.583 and 0.329, respectively, and both methods had numerous data points substantially deviating from the central data trend. Taken together, these data indicate that Circle-Map has higher sensitivity than CIRCexplorer2 and Circle_finder while maintaining a high precision.

## Conclusion

In conclusion, we have developed a new method for detection of circular DNA based on a full probabilistic model for aligning reads across the breakpoint junction of the circular DNA structure. Using this model in combination with our guided realignment procedure, we are able to accurately align even very short soft clips (> 4 nts). Using both simulated and real datasets, we have shown that this approach is both highly sensitive and precise - and significantly better than state-of-the-art methods from the circular DNA field and the related field of circular RNA detection, when applied to DNA. We expect our algorithm to obtain its maximum accuracy on circle enriched data. Our method may also be applicable to standard whole genome sequencing data, however this will required additional testing of the method. Furthermore, we note that our method is restricted to detect reads crossing the circular DNA breakpoints and hence cannot determine variation within DNA circles; this problem can be solved using methods designed to reconstruct the internal structure of the DNA amplifications [13].

We predict that Circle-Map will find widespread usage across the expanding field of circular DNA research spanning from healthy and cancerous human tissues to model organisms such as yeast, worm and mouse. Finally, we speculate that Circle-Map will likely also improve upon the state-of-the-art methods for detection of circular RNA.

## Availability and requirements

**Project name:** Circle-Map

**Project home page:** https://github.com/iprada/Circle-Map

**Operating system(s):** Platform independent

**Programming language: Python3.6**

**Other requirements:** None

**License:** MIT License

**Any restrictions to use by non-academics:** None

## Material and methods

### Circle-Map simulation tool

In order to create a synthetic benchmark dataset we created a circular DNA simulation tool, included in the Circle-Map package. Our tool requires the genome reference sequence indexed with SAMtools [14] and the number of reads to simulate, and it will produce the simulated FASTQ files together with a BED file indicating the chromosomal coordinates of the circular DNA. The procedure involves three parts to simulate circular DNA. First, to consider genetic variation not present in the linear reference genome, it will introduce base substitutions and indels with BBMap (https://sourceforge.net/projects/bbmap/). Then, it will select the circle coordinates and the length from a uniform distribution (default range: 150–10,000 nts) and sample the reads from the mutated genome. To generate all kinds of ordinary and circular DNA candidate reads our tool begins by defining the start and end read alignment coordinates and the insert size generated from a user defined normal distribution. Then, if the coordinates directly overlap the defined breakpoint coordinates, It will obtain both anchors of the read and join them together to generate the split read. Likewise, if the reads do not overlap the breakpoints but the insert within the paired reads spans the circle breakpoint it will generate a discordant read pair by sampling both reads from each side of the breakpoint. If any of the above mentioned conditions are not met, Circle-Map will sample a regular concordant read pair. Finally it will introduce instrument specific sequencing errors on the reads with ART [15].

### Simulated data

We used Circle-Map Simulate to generate 13,097 circular DNA from the canonical chromosomes of the hg38 version of human genome, excluding the gap regions,downloaded from the UCSC genome browser [16] on the 1st of February, 2019. We simulated the circular DNA with a circle length distribution ranging from 150 to 10,000 nts. Altogether, we generated 2 × 10007326 paired end reads with a normally distributed insert size (mean = 300, s.d = 25) and a read length of 100 nts. We introduced indels and base substitutions on the reference at rates of 0.0001 and 0.001 respectively. Then, we generated reads with an Illumina HiSeq 2500 error profile without masking any bases (−nf option set to 0) and leaving the rest of the parameters as suggested by the ART authors.

We aligned the simulated reads to the canonical chromosomes of the canonical hg38 assembly using BWA-MEM [17] (v0.7.17-r1188) without modifying the mapping qualities of the supplementary alignments (−q option turn on) and we used SAMtools [14] (v1.9) to perform all the post processing step of the alignment files. In order to remove low quality alignments, we removed split reads where any of the split anchors had mapping qualities below 20 for both segments for CIRCexplorer2 [10] (v2.3.5) and Circle_finder (version hash in github: ca7c0f2), and we implemented the same filter internally on Circle-Map. Afterwards, we executed CIRCexplorer2, Circle_finder and Circle-Map leaving all the parameters as default and we removed the circular DNA containing less than two breakpoint reads, requiring Circle-Map to contain at least 1 split read in the set of the 2 breakpoint reads. Finally, as benchmark criteria for the simulated dataset, we used sensitivity, defined as the fraction of predicted circular DNA overlapping the simulated set by a fraction of 0.95, and precision, defined as the fraction of simulated circular DNA overlapping the predicted set by a fraction of 0.95.

To evaluate the performance of Circle-Map on the short circles, we generated 2 × 200040 Illumina paired-end reads with a read length of 100 nts. All in all, we generated 5384 circles at 30X coverage with a length ranging from 150 to 350 nts. We considered sequencing errors and genetic variants not present in the reference genome as described for the 30X simulated data, and we used the same circular DNA calling strategy as described above.

### Real data

We downloaded a dataset (SRA ID: SRR6315430) from human muscle where the circular DNA was enriched prior to sequencing using the Circle-Seq [18] procedure. Briefly the Circle-Seq procedure purifies DNA in 4 steps: isolation of the DNA by column separation, elimination of the residual linear DNA using exonuclease digestion followed by rolling circle amplification and paired sequencing on an Illumina HiSeq 2500 machine. In total, the dataset consisted of 2 × 12829402 paired reads with a length of 100 nts. We used prefetch and fasterq-dump from the SRA toolkit (https://github.com/ncbi/sra-tools v2.9.1) to download and convert the SRA files to FASTQ, respectively. We used the same strategy as described for the simulated data to detect the circular DNA in the real dataset. As performance measure for the real dataset, we first plotted a histogram representing the number of circular DNA against the percentage of bases covered within the detection coordinates of every circular DNA. We set the bins of the histogram to intervals of 10%. Finally, as a second performance measure, we plotted the number of discordant reads and split reads against the mean sequencing coverage within the circular DNA coordinates, and we calculated the Pearson’s correlation coefficient as implemented in SciPy [19].

### Comparison of computational resources

All the computational comparisons were done by reserving a single computing node containing 128 GB of RAM memory and an Intel(R) Xeon(R) CPU E5–2670 0 @ 2.60GHz processor with 16 cores. The node was connected to a BeeGFS storage system via Infiniband 4X QDR.

### Size distribution of the circles found in human muscle

In order to compare the circular DNA size distribution in human muscle described in the original study [3] with the size distribution described in Circle-Map we downloaded the Supplementary file 1 from the original study and processed the detected DNA circles with the same procedure as the one we used for Circle-Map: all the circles that did not contain at least 2 breakpoint reads with at least 1 split read between those were excluded from the analysis.

### Circle-Map default circular DNA filtering

Circle-Map implements hard filters at the alignment and interval level to control for circle detection errors not accounted by the probabilistic model. At the alignment level, Circle-Map removes BWA-MEM flagged discordant reads pairs and split read primary alignments with mapping qualities below 20. The secondary alignments of the split reads with mapping qualities below 20 are not removed, but remapped using Circle-Map realignment algorithm in order to consider them as supportive for circular DNA. Furthermore, under Circle-Map realignment model, realigned reads with an edit distance greater than 0.05 as fraction of the read length are not considered. Circle-Map applies two hard filters to the detected intervals. First, Circle-Map removes circular DNA with allele frequencies smaller than or equal to 0.1, calculated as the number of split reads crossing the breakpoint divided by the mean sequencing coverage at the breakpoint nucleotides. Finally, to avoid redundant circular DNA identifications, circular DNA overlapping reciprocally by a fraction of 0.99 are combined into one interval.

### Circle-Map default output

Circle-Map will provide a tab separated file containing all the detected circular DNA. For every circular DNA it will provide information containing the circular DNA mapping and information about the sequencing coverage in the circular DNA coordinates. Regarding the mapping based metrics, Circle-Map provides the mapping coordinates (chromosome, start and end), breakpoint read support (discordant and split reads) and a circular DNA mapping score, calculated by multiplying the length of the split read by its mapping probability, and summing over all the scores for the split reads supporting a circle. In relation to the sequencing coverage information, Circle-Map provides the mean sequencing coverage within the detection coordinates, standard deviation of the sequencing coverage, the fraction of circular DNA bases not covered by sequencing reads and, finally, the sequencing coverage increase upstream and downstream of the detection coordinates. We calculated the increase in coverage as the ratio between the number of reads aligned 100 nts inside the breakpoint boundaries of the circular DNA and the same region extended 200 nts downstream (for the left part of the breakpoint) and upstream (for the right part of the breakpoint).

### Code availability

Circle-Map is implemented in python3 with the computational bottlenecks accelerated through multiprocessing and just-in-time compilation with Numba [20]. Circle-Map can be easily installed through the Python Package Index and the bioconda project [21]. The source code is released under the MIT license and it is freely available at https://github.com/iprada/Circle-Map.

## Data Availability

The sequencing dataset from human muscle are publicly available under the Sequence Read Archive with the ID: SRR6315430

## Abbreviations

Gb: Gigabytes
Indels: Insertions and deletions
Nts: Nucleotides
PSSM: Position-Specific Scoring Matrix
SNV: Single Nucleotide Variation
